# A Review on the Anti-Inflammatory Activity of Pomegranate in the Gastrointestinal Tract

**DOI:** 10.1155/2013/247145

**Published:** 2013-03-14

**Authors:** Elisa Colombo, Enrico Sangiovanni, Mario Dell'Agli

**Affiliations:** ^1^Department of Pharmacological and Biomolecular Sciences, Università degli Studi di Milano, Via Balzaretti 9, Milano, Italy; ^2^Research Centre for Characterization and Safe Use of Natural Compounds-G. Galli, Università degli Studi di Milano, Via Balzaretti 9, 20133 Milano, Italy

## Abstract

Several biological activities of pomegranate have been widely described in the literature, but the anti-inflammatory effect in the gastrointestinal tract has not been reviewed till now. The aim of the present paper is to summarize the evidence for or against the efficacy of pomegranate for coping with inflammatory conditions of the gastro-intestinal tract. The paper has been organized in three parts: (1) the first one is devoted to the modifications of pomegranate active compounds in the gastro-intestinal tract; (2) the second one considering the literature regarding the anti-inflammatory effect of pomegranate at gastric level; (3) the third part considers the anti-inflammatory effect of pomegranate in the gut. *In vivo* studies performed on the whole fruit or juice, peel, and flowers demonstrate antiulcer effect in a variety of animal models. Ellagic acid was the main responsible for this effect, although other individual ellagitannins could contribute to the biological activity of the mixture. Different preparations of pomegranate, including extracts from peels, flowers, seeds, and juice, show a significant anti-inflammatory activity in the gut. No clinical studies have been found, thus suggesting that future clinical studies are necessary to clarify the beneficial effects of pomegranate in the gastrointestinal tract.

## 1. Introduction

*Punica granatum* L. (pomegranate, Family Lythraceae) is a deciduous tree distributed throughout the world. Pomegranate (PG) fruit, in the form of extract of juice, is widely promoted to consumers since the nineties as medicinal food in the United States and Europe; as a consequence, a large number of pomegranate-containing products have recently been introduced into the USA and European market and are widely sponsored as healthy products.

PG is used in the traditional medicine of different Asian cultures for the treatment of a variety of ailments. In India, Tunisia, and Guatemala, dried PG peels are decocted and employed both internally and externally as astringents and germicides and used for treating aphthae and diarrhoea. In Ayurvedic medicine the plant, described under its Sanskrit name “dadima” (fruit), is considered as a “blood purifier” and used to cure parasitic infections (for a review, see [1]), and the decoction of the root is considered helpful against fevers and chronic debility due to malaria. *Punica granatum* L. fruit rind is traditionally used in the eastern province of Orissa (India), an area endemic for both *Plasmodium falciparum* and *Plasmodium vivax*, against malaria; in this regard, the beneficial effect of the fruit rind of PG for the treatment of malaria has been recently ascribed to the antiparasitic activity [2] and to the inhibition of the proinflammatory mechanisms involved in the onset of cerebral malaria [3].

The biological activity of PG has been widely investigated, including *in vitr*o, *in vivo,* and clinical studies. The beneficial effects are mostly the cardiovascular protective role, neuroprotective activity, hypoglycemic effect, and anticancer properties, in particular against prostate, colon, and breast cancer; the anticancer effect is limited only to *in vitro* and animal studies [1, 4, 5]. 

The gastrointestinal tract represents an important barrier between the human hosts and microbial populations. One potential consequence of host-microbial interactions is the development of mucosal inflammation, which can lead to gastritis and ulcer. 

Gastritis is defined as inflammation of the gastric mucosa. There are several etiological types of gastritis, differing for clinical manifestations and pathological features. Gastritis can be caused by endogenous and exogenous factors, including acid, pepsin, stress, and noxious agents such as alcohol, nonsteroidal anti-inflammatory drug (NSAIDs), *Helicobacter pylori* (*H. pylori*) infection, and smoking. *H. pylori*, a Gram-negative bacterium that colonizes the stomach of humans and primates, is the most responsible pathogen for these inflammatory processes. *H. pylori* infection in humans represents a serious public health concern: the WHO classifies this bacterium as a Type 1 carcinogen. The clinical course of *H. pylori* infection is highly variable and is influenced by both microbial and host factors. The pattern and distribution of gastritis strongly correlate with the risk of clinical duodenal or gastric ulcers, mucosal atrophy, gastric carcinoma, or gastric lymphoma. It has been demonstrated that gastric epithelial cells, after *H. pylori* infection, show higher levels of cytokines including IL-1*β*, IL-6, TNF-*α*, and IL-8, a potent neutrophil-activating chemokine that apparently plays a central role in gastric diseases [6, 7]. *H. pylori* strains carrying the Cag-PAI (Cag Pathogenicity Island) induce a far stronger IL-8 response than Cag-negative strains, and this response depends on activation of NF-*κ*B and the early-response transcription factor Activating Protein-1 (AP-1) [8]. IL-21 is constitutively expressed in gastric mucosa as well and is more abundant in biopsy specimens from *H. pylori*-infected patients [9]. 

Inflammatory bowel diseases (IBDs), among which Crohn's disease (CD) and ulcerative colitis (UC), are the most common inflammatory-related diseases in the gut; IBDs occur in response to genetic or environmental factors and are characterized by the uncontrolled response of the intestinal immune system against the normal enteric microflora, leading to abdominal pain and chronic diarrhoea. All components of the gut, including the epithelial barrier, the mucosal immune system, and stromal/supportive cells, participate in the intestinal immune response. Immune and nonimmune cells, that is, epithelial, endothelial, mesenchymal, and nerve cells, exchange regulatory signals via the production of mediators (cytokines, growth factors, adhesion molecules, etc.), which facilitate and amplify cell interactions and inflammation [10]. 

Epithelial cells, in response to a proinflammatory stimulus, that is, TNF*α* and bacteria, release several cytokines and induce the expression of NF-*κ*B related genes such as cyclooxygenase-2 (COX-2), inducible nitric oxide synthase (iNOS) and metalloprotease-9 (MMP-9) [11]. The integrity of the epithelial barrier is reduced thus favouring pathogen infections. NF-*κ*B is highly involved in the control of the transcription of the inflammatory mediators. 

Anti-inflammatory properties of PG and its major components have been widely described in the literature (for a review, see [12]). For example, cold pressed PG seed oil has been shown to possess anti-inflammatory activity since it inhibited *in vitro *both cyclooxygenase and lipoxygenase enzymes. In addition, the acetone extract of whole PG fruit inhibited phosphorylation of several cytokines released by UV-B-irradiated keratinocytes, and the mechanism underlying this effect was found to be NF-*κ*B-dependent [13]. 

Although some papers describe the beneficial effects of this fruit against gastro-intestinal inflammation, surprisingly this has not been reviewed till now. 

The aim of the present paper is to summarize the evidence for or against the efficacy of PG for addressing inflammatory conditions of the gastro-intestinal tract.

The paper will be divided in three parts: (1) the first one will be devoted to the modifications of PG active compounds in the gastro-intestinal tract, with particular attention to the intestinal metabolites; (2) the second one considers the literature regarding the anti-inflammatory effect of PG and individual compounds at gastric level; (3) the third part considers the anti-inflammatory effect of PG and individual compounds in the gut, taking into account also the main metabolites which are formed by microbial biotransformation after PG consumption. 

## 2. Pomegranate Composition and Metabolism in the Gastrointestinal Tract

PG has been shown to contain more than 100 different phytochemicals, and a substantial part of them contributes to the antioxidant activity elicited by the extracts [14]. However, several variables, such as the harvesting season, soil, and the kind of extract, can deeply affect chemical composition and consequently the biological activity. 

Ellagitannins (ETs) and anthocyanins (ANs) represent the most abundant polyphenols in PG juice. ETs constitute a complex class of polyphenols characterized by one or more hexahydroxydiphenoyl (HHDP) moieties esterified to a sugar, usually glucose. ETs content in PG juice is around 1500–1900 mg/L [15]. This part of the fruit contains several ETs typical of PG such as punicalagins, and other minor tannins including punicalin and gallagic acid. Punicalagins are unique to PG, and possess a molecular weight greater than 1000. During the juice processing, the whole fruit is pressed, and ETs are extracted into PG juice in significant amount, reaching levels over 2 g/L [15]. PG fruit contains also a small amount of free ellagic acid (EA), and it has been estimated to be around 15 mg/L in fresh arils [15]. 

ETs are quite stable under the physiological conditions of the stomach. The acid conditions (HCl, pH 1.8–2.0) and the gastric enzymes do not hydrolyse the native ETs to ellagic acid, and no degradation of ETs has been observed. The stomach seems to be a location for the absorption of free EA, but ETs are not absorbed [16]. PG ETs release EA in the gut, and this compound is poorly absorbed in the small intestine; conversely, EA is largely metabolized by human gut microflora in the intestinal lumen into urolithins, such as urolithins A and B, and urolithin-8-methyl ether [17, 18]. These metabolites reach relevant plasma concentrations (3–5 *μ*M) after PG juice consumption [19]. The absorbed metabolites are conjugated with glucuronic acid and/or methylated to give ether derivatives [16].

In addition to ETs, PG fruit is an important source of ANs as well; these include the 3-glucosides and 3,5-diglucosides of delphinidin, cyanidin, and pelargonidin [15, 20]. The total amount of ANs in PG fruit is higher in fresh arils than in the frozen ones (306 versus 172 mg/L, resp.), and in single-strength commercial juice than in commercial juice from concentrate (387 versus 162 mg/L, resp.) [15]. 

The pH of the stomach (1-2) ensures that ANs are maintained as the flavylium cation, which is the most stable form of ANs. The stability of ANs under the gastric conditions has been confirmed by *in vitro* studies [20, 21]. Conversely, the neutral pH of the small and large intestines makes ANs much less stable, and these molecules are converted into a variety of metabolites [22]. Several studies reported that exposure of different ANs to gut microflora resulted in rapid deglycosylation and demethylation to the corresponding aglycones. The aglycones were unstable at neutral pH and rapidly degraded to their corresponding phenolic acids and aldehydes through cleavage of the C-ring. Similar results were obtained after incubation of free and acylated ANs with human faecal microbiota [23, 24]. It has been proposed that the decrease of anthocyanin concentration after pancreatin bile salt digestion (as a simulation of small intestine digestion) could be partially explained by the transformation of the flavylium cation to the chalcone at the neutral pH [20]; however these hypotheses need to be confirmed by *in vivo* studies.

## 3. Effect of Pomegranate in Gastric Inflammation

There are no clinical studies in the literature investigating the beneficial effect of PG in the stomach. The anti-inflammatory activity of PG at gastric level has been evaluated mainly by *in vivo* studies, and few *in vitro* studies deal with the anti-*H. pylori* activity of PG extracts and individual compounds. For a better comprehension of the effects of PG in modulating gastric inflammation, in the following paragraph the *in vivo* studies have been organized according to the inflammatory challenge applied to induce gastritis in animal models.

### 3.1. *In Vitro* Studies

A small number of studies report that PG is able to treat *H. pylori* infection. Methanol peel extract of PG exhibited a remarkable anti-*H. pylori* activity *in vitro*, as shown by the size of inhibition zone in the disk diffusion method, which was comparable to the reference compound metronidazole (MIC 8 *μ*g/mL) [25]. In another study the methanolic extract of PG fruit rind exhibited high activity against *H. pylori* strains (39 mm inhibition zone for 100 *μ*g disc^−1^) in comparison with other plant extracts [26]; in particular eight among nine PG cultivars showed high activity against this bacterium (mean of inhibition zone diameter ranging from 16 to 40 mm at 50 *μ*g disc^−1^). Strong anti-*H. pylori* activity of PG ethanolic extract from pericarp has been also documented, and the highest zone of inhibition (16.5 mm) was found at 2.5 mg disc^−1^ [27].

Indeed, further studies with PG extracts alone and combined with drugs commonly used for *H. pylori* eradication should be carried out before considering PG as effective antimicrobial agent.

### 3.2. Animal Studies

*Ethanol-Induced Gastritis*. Ethanol-induced ulcers are the result of a direct effect of ethanol on gastric mucosa, mostly due to its capacity to induce necrosis of superficial gastric epithelial cells and erosion [28]. An *in vivo* study showed that PG fruit rind methanolic extract showed a significant reduction in the ulcer index (UI) in ethanol-induced gastritis in rats; the extract, when tested at 250 mg/kg and 500 mg/kg, inhibited UI by 21% and 63%, respectively, whereas the reference compound ranitidine (50 mg/kg) showed 51% inhibition. Rats treated with 500 mg/kg of PG extract were also protected from intraluminal bleeding [29]. In another study, the hydroalcoholic extract (methanol : water 80 : 20) from dried flowers of PG significantly reduced the UI (−87.5% at 980 mg/kg), whereas the half dose (490 mg/kg) and omeprazole (20 mg/kg) exhibited 65% and 49.6% inhibition, respectively [30]. 

Some biochemical parameters, such as superoxide dismutase (SOD), catalase, tissue lipid peroxidation, and glutathione peroxidase (GSH-PX), were reduced in animals treated with acetylsalicylic acid and returned at the basal levels in animals that received the fruit rind methanolic extract [29]. PG tannins significantly inhibited ethanol-induced mucosal injury in a dose-dependent manner, and the effect was ascribed to the decrease of lipid peroxidation as well as NO levels and to the modulation of both SOD and GSH-PX in gastric mucosa [31].

Gharzouli et al. demonstrated that aqueous extract of PG peel (AEP) shows a gastroprotective effect in rats with ethanol-induced gastric lesions [32]. The simultaneous administration of ethanol and AEP significantly decreased gastric lesions and UI (from −53.7% to −76.9%). 

Among the pure compounds, Beserra et al. demonstrated that the oral pretreatment with EA (3, 10, and 30 mg/kg) significantly reduced gastric injury by 59, 79, and 70%, respectively, whereas the inhibitory effect by ranitidine (50 mg/kg) was −83% [33]. 

Nonprotein sulfhydryls (NP-SH) are antioxidant compounds involved in the maintenance of gastric integrity. They control the cascade of inflammatory cytokines and promote detoxification and excretion of ROS produced mainly by noxious agents and stress. Pretreatment with EA (3 and 10 mg/kg) significantly increased the NP-SH content in rats thus suggesting a protective role of EA against ethanol-induced gastritis [33]. 

TNF-*α* released by monocytes/macrophages plays a key role in initiating the cascade of other proinflammatory cytokines, including IL-1*β*, IL-6, and IFN-*γ*, which are important biomarkers in gastric inflammation. Rats subjected to ethanol-induced ulcer after treatment with EA (10 mg/kg) showed levels of TNF*α* significantly lower than the ethanol-treated group (11.1 ± 1.1 versus 6.2 ± 1.0 pg/mL, resp.) [33].

NO is considered to be one of the most important defensive endogenous agents in the gastric mucosa. Pretreatment with inhibitors of NO synthase such as L-NAME has been demonstrated to worsen the ethanol-induced ulcer. Rats pretreated with L-NAME increased the severity of the gastric lesions induced by ethanol, whereas treatments with L-arginine, in the absence or in the presence of EA, significantly attenuated the deleterious effect of L-NAME (−22% and −19.6%, resp.). When EA was coadministrated with L-arginine, the gastroprotective effect was found 2-fold higher (−39%) with respect to EA alone [33].

*NSAIDs-Induced Gastritis. *Nonsteroidal anti-inflammatory drugs (NSAIDs), such as acetylsalicylic acid, indomethacin, and ibuprofen, are commonly used as analgesics in many acute and chronic inflammatory conditions. Acetylsalicylic acid interferes with gastric protective mechanisms, such as mucus and bicarbonate secretion, surface epithelial hydrophobicity, and mucosal blood flow. Acetylsalicylic acid mainly interferes with the biosynthesis of cytoprotective prostaglandins through the inhibition of cyclooxygenase (COX); this effect results in an over production of leukotrienes and other products of the 5-lipoxygenase pathway.

PG hydroalcoholic extract (methanol 70%) from powdered rind showed a significant UI reduction in rats treated with 400 mg/kg acetylsalicylic acid. The inhibitory effect showed by PG extract, at 250 and 500 mg/kg, was −22.4% and −74.2%, respectively, whereas ranitidine (50 mg/kg) showed 44.7% inhibition [29]. In another study, the hydroalcoholic extract (methanol : water 80 : 20) obtained from powdered flowers of PG, when tested at 980 mg/kg and 490 mg/kg, inhibited ulceration by 83.9% and 73.8%, respectively, thus suggesting that PG flowers contain compounds with a dose-dependent gastric protective effect [30]. 

Prostaglandins produced by COX-1, mainly PGI_2_ and PGE_2_, are essential for gastric mucosa protection. The ulcerogenic effect of NSAIDs seems to be related to the inhibition of endogenous prostaglandin synthesis, although it has also been established that indomethacin modifies other protective mechanisms of the gastric mucosa, including gastric secretion and the permeability of the gastric mucosal barrier [34]. Indomethacin is known to increase leukotriene C_4_ and reduces PGE_2_ levels. Enhancement of leukotriene synthesis results in damaging effects, which may induce mucosal vasoconstriction, and enhances NSAIDs-induced injury [35]. 

Hydroalcoholic (methanol : water 80 : 20) extract from powdered flowers of PG (980 mg/kg) showed significant prevention of gastric lesions in indomethacin-treated rats, higher inhibition of ulceration (83.9%), and reduced gastric acid secretion in comparison with omeprazole 20 mg/kg (69.5%). Although pure compounds were not tested during this study, the authors suggest that the anti-ulcerogenic activity of the extract may be due to the presence of saponins, tannins, and flavonoids [30].

The treatment with EA (3, 10, and 30 mg/kg) significantly decreased UI by 82, 74, and 77%, respectively, whereas the inhibitory effect, after treatment with cimetidine (100 mg/kg), was 88% [33]. Leukotriene B_4_ (LTB_4_) contributes to the NSAID gastric injury by promoting intense chemotaxis of the neutrophils and leukocyte adherence to the vascular endothelium. Plasma levels of LTB_4_ were also reduced with the treatment of EA (3, 10, and 30 mg/kg), but levels of prostaglandin E_2_ (PGE_2_) in gastric mucosa were not affected [33]. In another study, treatment with EA or omeprazole for 3 days significantly upregulated the mucosal PGE_2_ level by 2.0- and 1.6-folds, with respect to the untreated group [36]. In the same study, indomethacin-administered mice increased mucosal myeloperoxidase (MPO) activity on the third day. Treatment with EA (7 mg/kg once daily for 3 days, orally) and omeprazole significantly reduced MPO activity by 77.9% and 68,1%, respectively. Ulceration significantly depleted the expression of gastric COX-1. In this study, COX-2 expression was not significantly modified by indomethacin treatment; conversely, EA significantly increased COX-1 and COX-2 expression in the ulcerated group [36].

The same study investigated the effect of EA on indomethacin-induced cytokines release in the gastric mucosa. Indomethacin administration increased the release of several cytokines, including TNF-*α* (1.8-fold), IL-1*β* (1.9-fold), and decreased IL-4 (2.3-fold), IL-10 (1.2-fold), VEGF (1.7-fold), EGF (1.6-fold), and HGF (1.5-fold), whereas IL-6 secretion was not affected. EA (7 mg/kg) significantly reduced the proinflammatory cytokines (TNF-*α*, IL-1*β*, and IL-6) release by 1.9-, 1.5-, and 1.6-folds, respectively; a significant increase was seen for the anti-inflammatory cytokines IL-4 and IL-10 levels and growth factors (VEGF, EGF, and HGF) levels. When EA was administrated to the animals, it was able to decrease TNF*α*, IL-1*β*, and IL-6 release, and an increase of IL-4 and IL-10 was observed. When EA was given following COX inhibitor pretreatment (celecoxib, NS398), the protective effect was partially missed [36].

*Acetic Acid-Induced Gastritis.* No studies dealing with the effect of PG extracts on acetic acid-induced gastritis are present in the literature, whereas some studies on the pure compound EA occur. Treatment with EA (3, 10, and 30 mg/kg) for 14 days did not reduce the ulcerated area caused by application of acetic acid, but showed a significant reduction in the ulcer thickness (−21,3%, −25,6%, and −26,2%, resp.) in comparison to the vehicle group. Cimetidine significantly reduced the ulcerated area and the thickness of the ulcer by 33.4% [33].

In acetic acid ulcer model the plasma levels of TNF*α* were significantly lower in animals treated with EA at the dose of 3 mg/kg with respect to the control group (8.8 versus 19.3 pg/mL, resp.) [33]. IL-4 is an anti-inflammatory cytokine able to exacerbate, if excessively released, the inflammatory injury in the stomach. It has been demonstrated that ulceration drastically increased the plasma levels of IL-4 by 6-fold, and co-administration with EA significantly suppressed the increase by 65.0%, 60.6%, and 44.2% at 3 mg/kg, 10 mg/kg, and 30 mg/kg, respectively [33]. In a similar fashion, ulceration increased the plasma levels of IL-6 by 12.5-fold as compared to the control group, and EA significantly inhibited IL-6 levels by 75%, 73%, and 48% at 3 mg/kg, 10 mg/kg, and 30 mg/kg, respectively. The same trend was observed for IFN-*γ*. Acetic acid ulceration significantly increased the levels of IFN-*γ* by 7.2-fold, as compared to the control group. Pretreatment with the three concentrations of EA significantly inhibited the IFN-*γ*-induced gastritis by 83.8%, 63%, and 54%. IL-1*β*, IL-10, and VEGF levels were undetectable on the 14th day of ulcer induction in this model of gastritis [33].

*Pylorus-Ligated Gastritis. *The hydroalcoholic (methanol : water 80 : 20) extract of PG flowers demonstrated significant reduction in volume of gastric acid secretion and total acidity of gastric juice in rats with pylorus-ligated gastritis, whereas pH of gastric juice increased. Administration of the extract (980 mg/kg) was found to reduce the volume of gastric acid up to 40.0% as compared to the control group; the reduction of the gastric volume (−24.4%) was present also when the extract was given at the lower dose (490 mg/kg) [30]. In another study, PG tannins significantly increased the secretion of adherent mucus and free mucus, but did not affect the free acidity, total acidity, gastric juice volume, and gastrin pepsin activity induced by pylorus ligation [31].

PG hydroalcoholic extracts (ethanol 50%) from peel (100 mg/kg), rind (500 mg/kg), and seed (500 mg/kg) significantly decreased the mucosal injury at the 6th day of treatment, showing the antiulcer effect [37]. In the same study, EA reduced gastric acid secretion to 65–70% in pylorus-ligated rats after intraperitoneal administration at 5 and 10 mg/kg, although the effect was not statistically significant. On the contrary, acidity was significantly reduced by EA (5 mg/kg) treatment [38].

*Gastric Ischemia/Reperfusion.* Gastric ischemia induced for 20 minutes by bleeding from the carotid artery produced a marked reduction of gastric mucosal blood flow (GMBF) up to 40–50% of basal values. The GMBF then increased over basal values during 15 minutes reperfusion, reaching a maximum of 140–160% before gradually returning to the basal levels. Rats treated with EA (6 mg/mL) or SOD (15000 unit/kg/hr) did not show any differences in GMBF with respect to the control group [39].

Reduction of hemorrhagic lesions following gastric ischemia/reperfusion significantly started at 3 mg/mL of EA. The increased levels of lipid peroxidation induced by ischemia/reperfusion was significantly inhibited when animals were pretreated with either EA (6 mg/mL) or SOD (15000 unit/kg/hr), the inhibition being 78.3% and 82.6%, respectively [39].

In another study, topical application of NH_4_OH (60 mM) produced a persistent reduction of gastric potential difference in the stomach made ischemic by bleeding and resulted in hemorragic damage 1 hr later. The development of gastric lesions induced by NH_4_OH plus ischemia was dose-dependently inhibited by preexposure of the mucosa to EA (1–6 mg/mL), and a significant effect was observed at concentrations above 3 mg/mL [30].

*Stress-Induced Gastritis.* No studies investigating the effect of PG extracts against stress-induced gastritis have been published so far. Murakami et al. report that EA significantly inhibited the occurrence of stress-induced gastric lesions at 5 mg/kg [38], whereas the effect of the other pure compounds, including ETs and ANs, was not investigated.

## 4. Effect of Pomegranate in Intestinal Inflammation

Anti-inflammatory properties of PG and its major components have been widely described in the literature (for a review, see [12]). However only few papers reported the effect on intestinal inflammation, and, surprisingly, no clinical studies are present in this regard. In the following paragraph, the *in vitro* and *in vivo* anti-inflammatory effect of *Punica granatum *L. extracts and individual compounds at the intestinal level will be reviewed and discussed. 

### 4.1. *In Vitro* Studies

*PG Juice and Peel/Husk Extract*. The anti-inflammatory effect of PG on an *in vitro* intestinal model was firstly described in 2006 in a study investigating the molecular mechanisms underlying its antitumoral properties [40]. It was demonstrated that pretreatment of a colon cancer cell line with commercial PG juice (6–50 *μ*g/mL), total pomegranate tannins (TPT, 30–200 *μ*g/mL), and punicalagin (25–200 *μ*g/mL) inhibited AKT activity, NF-*κ*B activation, and COX-2 expression induced by TNF*α* [40]. PG juice showed the highest activity on these parameters with respect to TPT and the pure component, indicating that more than a single bioactive compound contributes to the biological activity exerted by the extract. Using a different cellular model (human intestinal Caco-2 cells), Romier-Crouzet et al. demonstrated that the pretreatment with a polyphenolic aqueous extract from PG peels (PomH) reduced many inflammatory mediators [41]. The extract, at the concentration of 50 *μ*M (expressed as total phenols), significantly inhibited the secretion of the chemokine IL-8 and the production of NO upon stimulation by IL-1*β* or a mixture of proinflammatory stimuli. It also exhibited an inhibitory effect on the secretion of PGE_2_ by COX-1 as well as COX-2 in response to IL-1*β* stimulation [41]. Authors explained this anti-inflammatory activity with the inhibition of NF-*κ*B activation and ERK-1/2 phosphorylation; both systems are implicated in IL-8, NO, and PGE_2_ expression [41]. The same group demonstrated that pretreatment with PG husk extract (PomH, 100 *μ*g/mL) and its pure ellagitannin punicalagin (50 *μ*M) could modify transcription levels of the genes encoding for IL-6 and MCP-1 in differentiated Caco-2 cells treated with a mixture of proinflammatory stimuli (IL-1*β*, TNF-*α*, IFN-*γ*, and LPS) [42]. No effect was seen in the absence of proinflammatory conditions and on IL-8 expression, while PomH as well as punicalagin decreased the secretion of IL-8, IL-6, and MCP-1 [42]. A direct interaction between punicalagin and cytokines was also demonstrated, thus suggesting that PomH ETs could interact with the proinflammatory cytokines in the gut lumen, decreasing the intercellular communication and limiting the local and systemic inflammation [42].

*PG Metabolites and Urolithins*. Phenolic compounds contained in PG extracts are of particular importance during intestinal inflammation. In fact, it was demonstrated that only a very small percentage of polyphenols ingested with diet are absorbed in the small intestine, while the majority of them remain in the gut lumen, where they counteract and are metabolized by gut microbiota. In this regard, it was shown that, after a digestion *in vitro*, ANs, phenolic compounds, and vitamin C present in PG juice diminish their solubility, compromising the amount of compounds that can be absorbed by intestinal cells and their bioavailability [20]. However, this permanence of phenolic compounds ingested with PG in the intestinal lumen enhances the interactions with gut microbiota. Many scientific evidence points out the importance of human gut microbiota toward health improvement and the genesis of various diseases. In fact beneficial bacteria species, as *Bifidobacterium* and *Lactobacillus*, constitute an important barrier against the overgrowth of external and internal pathogenic strains, whose prevalence causes chronic and acute bowel diseases and has been associated with aging and cancer [43]. For this reason, the modulation of gut microbiota by dietary supplements and food intake is of key importance to the maintenance of physiological balance. In this regard, a commercial PG dietary supplement derived from extraction of residual material from juice production (PomX) and punicalagin demonstrated a selective toxicity toward intestinal pathogenic bacteria as compared to probiotic bacteria [44]. In particular, PomX, as well as punicalagin and EA, significantly inhibited *Clostridium* and *Staphylococcus aureus* growth. Punicalagin showed an inhibitory effect also against the growth of *Escherichia coli *and *Pseudomonas aeruginosa* (IC_50_ 9.2 and 3.2 *μ*M, resp.). Probiotic lactobacilli were unaffected by PG components, while the positive effect on bifidobacteria was species-specific [44]. Possible explanations of the mechanisms underlying the antimicrobial effect against pathogenic bacteria have been hypothesized: (i) tannins could create stable complexes with proteins or with physiological metal ions essential for bacteria survival; (ii) polyphenols might decrease the pH of intestinal environment, favouring the growth of probiotic instead of pathogenic bacteria [44]. The long permanence (up to 56 hrs in the colon before excretion [45]) of PG ellagitannins and the interaction with gut microbiota lead to the formation of many metabolites, that could explicate different biological activities at this level. Through *in vitro* batch-culture fermentation system the effect of PomX extract and punicalagin on the growth of gut bacteria and the production of active metabolites was investigated. PomX, but not punicalagin, significantly enhanced the growth of total bacteria, as well as beneficial bifidobacteria and lactobacilli, being the effect in contrast with previous results [46]. The extract also increased the production of short chain fatty acids, acetate, butyrate, and propionate [46], compounds that are related to inhibition of preneoplastic proliferation and acceleration of conversion of cholesterol into bile acids [43]. As concerns the metabolism of PG ellagitannins, while punicalagin was not detected in the fermentation media at any collection time, after 10 hrs of incubation with PomX there was a pick of ellagic acid (EA) that disappeared when dibenzopyranones urolithins A, C, and D appeared [46]. Urolithin B was not detected in small intestine, suggesting that ETs metabolism occurs in the colon as well, and the formation of this compound is the last step of ETs metabolism. 

Both urolithins C and D as well as urolithin A demonstrated a high antioxidant activity *in vitro* (IC_50_ 0.16, 0.33, and 13.6 *μ*M, resp. for urolithin C, D, and A) [47], but what are the effects of PG metabolites on intestinal inflammation? In the first study, the antiphlogistic effects of EA (50 *μ*M) were evaluated in Caco-2 differentiated cells treated with a mixture of proinflammatory stimuli (IL-1*β*, TNF-*α*, IFN-*γ*, and LPS) on secretion and mRNA expression of different inflammatory biomarkers (IL-6, IL-8, MCP-1, and IL-10). EA decreased only MCP-1 and IL-8 secretion, but the effect was not statistically significant, and no effect on the mRNA levels of these cytokines was observed [48]. Nevertheless, in the same study, EA downregulated the transcription of some genes involved in intestinal inflammation, as STAT-3, a transcription factor responsible for the persistent activation of NF-*κ*B [48]. In another study, EA, urolithins A and B, and a mixture of them, at a concentration achievable in the intestinal lumen after PG consumption (10 *μ*M for EA and 40 *μ*M for urolithins), inhibited the growth of Caco-2 cancer cells, deactivating the ERK-signalling pathway [49]. Opposite results were obtained in human normal colon fibroblast cell line CCD18-Co treated with IL-1*β*, as a proinflammatory stimulus, and with different concentrations (1–10 *μ*M) of EA and urolithins A or B since neither urolithins nor EA was able to decrease the phosphorylation of p-ERK1/2 [50]. Myofibroblasts in the colon mucosa play an important role in the intestinal inflammatory response even by releasing PGE_2_. In the same study it was shown that urolithin A decreased PGE_2_ production, downregulating COX-2 and mPGES-1 expressions but not their enzymatic activity. Reduction of PGE_2_ production by urolithins could be explained by the inhibition of NF-*κ*B activation and p38 MAPK pathway [50]. No anti-inflammatory effects were seen with EA [50]. Authors hypothesized that the anti-flogistic activity demonstrated by urolithins might be due to a receptor-mediated effect, because no metabolites were found in cell media or intracellular extracts after incubation of cells with urolithins A, B or EA [50]. Using the same cellular model of human colon fibroblasts treated with IL-1*β* or TNF-*α* as proinflammatory stimuli and urolithins A (40 *μ*M), B (5 *μ*M) or EA (1 *μ*M), it was demonstrated that all these metabolites inhibit two critical processes of inflammatory response: fibroblast migration and monocyte adhesion. A mixture of urolithins A, B and EA completely abolished IL-1*β* induction by PGE_2_, and this activity was mainly due to urolithin A [51], in agreement with another previous study [50]. The effect was not maintained when TNF*α* was used as a stimulus, thus indicating that PG metabolites could explain their action by a challenge-dependent pathway [51]. PAI-1 is another molecule deeply associated with inflammatory status of the intestinal mucosa, whose expression is highly induced by IL-1*β* and TNF*α* and is strictly related to fibroblast migration. The metabolites mixture and urolithin A downregulated PAI-1 expression and exerted also a preventive effect inhibiting some factors of the PDGF family, known to be involved in cell migration [51]. During inflammation, the presence of cytokines such as IL-1*β* and TNF-*α* induces the expression of chemokines, in particular IL-8, and adhesion molecules (e.g., ICAM-1 and VCAM-1) in colonic subepithelial myofibroblasts, thus favouring monocyte infiltration and adhesion. The mixture of metabolites reduced monocyte migration inhibiting IL-8 secretion induced by TNF*α* and ICAM-1 and VCAM-1 levels after stimulation with IL-1*β* [51].

### 4.2. Animal Studies

There are two main standardized methods to produce an experimental animal model of IBD: (i) oral administration of dextran sulphate sodium (DSS) in drinking water; (ii) intracolonic administration of trinitrobenzene sulfonic acid (TNBS). The use of DSS method mimics the development of UC, while symptoms manifested after TNBS treatment present the clinical and morphological features of CD.

In a rat model of DSS-induced UC, oral administration of EA using colonic delivering microsphere (containing 1–10 mg/kg of EA) significantly reduced the severity of colonic lesions and the shortness of colonic length. This activity could be accounted by the antioxidative action of EA, as demonstrated by the decreased myeloperoxidase (MPO) activity and lipid peroxidation in the colonic mucosa of animals treated with EA microspheres [52]. These results were confirmed in another study, which showed that a PG hydroalcoholic extract (100–200 mg/kg) from flowers as well as the ellagic acid-rich fraction attenuated DSS-induced UC in mice, reducing colonic MPO activity and oxidative markers [53]. In addition to the antioxidant effect, another mechanism proposed for the anti-ulcerative properties of EA was the stabilization of mast cells. Mast cells play an important role in phlogistic processes by releasing many proinflammatory factors, as histamine. DSS administration in mice was also associated with an increase in histamine content in colonic tissue, indicating mast cell degranulation. Hydroalcoholic extract of PG (methanol : water 3 : 1) and its ellagic acid-rich fraction significantly attenuated the DSS-induced increase in the histamine level, suggesting a capacity to stabilize mast cells degranulation [53]. The molecular mechanisms underlying the anti-inflammatory activity of EA in the gut were clarified in a study devoted to the effect of this polyphenol on colon carcinogenesis [54]: EA (60 mg/kg), administered *per os* for 30 weeks in a rat model of colon cancer, reduced inflammatory status decreasing the expression and the production of COX-2, iNOS, IL-6, and TNF-*α* through the inhibition of NF-*κ*B system [54]. The same mechanism was confirmed in two studies analysing the anti-flogistic effect of EA in TNBS-induced colitis in rats: (i) in the first it was demonstrated that administration of EA (10-20 mg/kg) by gavage before and after induction of acute colitis diminished the severity and extension of the intestinal injuries and inhibited MPO activity, in agreement with previous studies [52, 53]. It was also shown that treatment with EA was able to reduce the neutrophilic infiltration and to increase the production of mucus in globet cells. All these anti-inflammatory effects might be explained by the reduction of the expression of COX-2 and iNOS, through the inhibition of NF-*κ*B-mediated transcriptional activation and preventing p38, JNK and ERK-1/2 MAPKs phosphorilation [55]; (ii) the second study analysed EA effect in a model of chronic colitis. Chronic oral administration of a commercial PG fruit extract (250–500 mg/kg) with or without the enrichment with EA (10 mg/kg) was able to reduce inflammatory symptoms and histological injuries, diminishing MPO activity, TNF-*α* production, and reducing COX-2 and iNOS expression through the inhibition of MAPKs and NF-*κ*B, but not PPAR-*γ*-signalling pathways [56].

In a rat model of UC induced by 2,4-dinitrochlorobenzene (DNCB) and acetic acid, intragastric administration of an aqueous extract from PG peel (200–800 mg/kg) relieved diarrheic and ulcerative symptoms, decreasing MPO activity, lipid peroxidation, IL-1*β*, and TNF-*α* [57]. 

In another study, the PG peel extract (PPE) demonstrated also to downregulate the expression of inflammatory genes (COX-2, IL-6, and IL-1*β*) in colonic and adipose tissues from obese mice fed with a high-fat diet [58]. The same extract possessed also a prebiotic effect, modulating gut microbiota toward bifidobacteria [58], which are bacteria showing high anti-flogistic activity in the intestinal tract [59]. Moreover, it was demonstrated *in vitro* that gut bacteria could contribute to the anti-inflammatory effects of PG extracts through their capacity to metabolize polyphenols to bioavailable and biological active urolithins [50]. To assess whether anti-flogistic properties seen with PPE were due to ETs content or to their microbiota-derived urolithins, a rat model of DSS-induced colitis was fed with PG peel extract (250 mg/kg) or urolithin A (15 mg/kg) for 25 days before administration of DSS. In rats fed with PPE and urolithins A, the number of bifidobacteria and lactobacilli increased, and the effect was maintained after DSS administration only in urolithin A-fed group. Both PPE and urolithin A increased also the count of *Clostridium* probiotic strains and maintained it after colitis induction, preventing the colonization and invasion of colonic tissue by pathogenic enterobacteria [60]. Treatment with PPE and urolithin A exerted the same anti-inflammatory effects, downregulating PGE_2_ production as well as iNOS induction and NO levels; however, in the case of PPE, these properties seemed not to be enough to protect the colonic architecture [60]. Another interesting aspect is the evaluation of metabolic fate of PPE after microbiota metabolism. In fact PG metabolism in rats with colitis was shown to be different from those without colon inflammation: the phenolic profile of faeces of PPE-fed rats showed the presence of ellagic acid and even punicalagin contained in PPE and traces of urolithin A, while only urolithins were found in faeces of healthy PPE-fed rats [60]. The low metabolism exerted by gut microbiota in an inflammation context is an important consideration studying the effects of phenolic compounds. In fact, as previously demonstrated, in the presence of an inflammatory status they can reach, without any metabolic transformation, the intestinal lumen and directly exert their anti-inflammatory activity. In conclusion, the anti-inflammatory properties shown by PG in the IBD model could be explained by a synergic activity of urolithins, the main active compound responsible for PG anti-inflammatory effects in healthy subjects, with ETs and EA; however, the increase in prebiotic bacteria by both PPE and urolithin A or both these hypotheses taken together cannot be excluded [60]. 

Pomegranate seed oil (PSO) is predominantly composed of triglycerides containing unsaturated fatty acids, as conjugated linolenic acids, oleic acid, linolenic acid, palmitic acid, and stearic acid. The major conjugated linolenic acid in PSO (representing 60 to 80% of total fatty acids) is punicic acid (PuA). Increasing evidence suggested that fatty acids with conjugated double bonds, such as PuA, exert beneficial effects in inflammation and some types of cancer [61, 62]. Different *in vivo* studies assessed the effects of PG seed oil and PuA on intestinal inflammation. Oral administration of PuA (800 *μ*g/mL) or PG seed oil (2%) before TNBS injection in a colitis rat model ameliorated ulceration status and tissue damage, limiting neutrophil activation and lipid peroxidation [63]. Molecular mechanisms underlying these anti-inflammatory effects were clarified by *in vitro* studies on human neutrophils. PuA (10 *μ*M) exerted a strong anti-oxidant activity, inhibiting TNF-*α*-induced ROS and NADPH oxidase production by neutrophils, through the inhibition of p47phox phosphorilation and p38 MAPK activation [63]. Treatment with PuA prevented also TNF-*α* and bacterial fMLP-induced neutrophil degranulation, resulting in reduced MPO release and lower tissue injuries [63]. In another study it was demonstrated that pretreatment with PuA (45–80 mg/die) to an IL-10^−/−^ IBD mice model ameliorated clinical symptoms, while it was not able to exert any effect if administered after IBD development [64]. PuA upregulated colonic PPAR-*δ* activation and suppressed TNF-*α* and MCP-1 expressions. Since effects of PuA disappeared in IL-10 and PPAR-*γ* or -*δ* double-knock-out mice, it was hypothesized that the pure compound exerted its action through PPAR-*γ* and -*δ* pathways [64]. This conclusion was in agreement with previous results demonstrating that PPARs represent important targets of dietary lipids, mediating the maintenance of intestinal homeostasis. Moreover, PPAR-*γ* expression results also in therapeutic benefits in the DSS colitis model [65], producing the same effects seen with PuA. PSO was shown to be effective also in a model of necrotizing enterocolitis (NEC). This disease is the major cause of morbidity and mortality in premature infants and is characterized by a dysregulation of inflammatory cytokines in the intestinal mucosa and an impaired production of mucins (as Muc2) and trefoil factors (Tff) by lamina propria. Treatment with PSO (1.5% w/w) of a neonatal model of NEC rats reduced the incidence and severity of necrotizing colitis, protecting the epithelial barrier and preserving the intestinal integrity [66]. PSO decreased IL-6, IL-8, IL-23, IL-12, and TNF-*α* mRNA levels as well and downregulated the inflammatory response in the developing intestinal mucosa [66].

*Antidiarrhoeal Effects.* Diarrhoea is one of the major symptoms of IBDs, mainly caused by inflammation and an increased intestinal transit leading to a reduction in water and electrolytes absorption. Two studies described the *in vivo* antidiarrhoeal effect of PG. Intraperitoneal injection of an aqueous extract of PG peels (100–400 mg/kg) as well as orally administration of a crude methanolic extract from PG rind (200–400 mg/kg) diminished diarrhoea induced by castor oil administration in a rat model [67, 68]. PG aqueous extract reduced in a dose-dependent way intestinal weight and motility, inhibiting also the spontaneous and acetylcholine-induced ileum contractions. Hypothesis formulated for this antidiarrhoeal activity included (i) an increase of reabsorption of water and NaCl, reducing intestinal motility and (ii) a decrease of cytosolic calcium, either by inhibiting Ca^2+^ influx or Ca^2+^ release from intracellular stores, resulting in relaxation of rat ileal smooth muscle [68]. On the other hand, PG methanolic extract exerted a potent antioxidant activity (IC_50_ 11.7 *μ*g/mL) and significantly scavenged nitric oxide (IC_50_ 12.5 *μ*g/mL), a molecule involved in the diarrhoeal effect induced by castor oil. Retardation of intestinal transit demonstrated by PG crude extract might be explained with an increased absorption of water and electrolyte, resulting in a diminished number of wet faeces [67].

## 5. Conclusions

Few *in vitro* studies have been performed with PG peel extracts to evaluate anti-*H. pylori* activity. These extracts are able to reduce significantly the growth of this pathogen, which is considered the aetiological agent mainly responsible for human gastritis. 

*In vivo* studies performed on the whole fruit or juice, peel, and flowers demonstrate high antiulcer effect in a variety of animal models. EA was found to be the main component responsible for this effect, although other individual ETs, which have not yet been studied, could contribute to the biological activity of the mixture. With the exception of EA, the effect of the pure compounds at the gastric level was not investigated; this should be carefully considered for the future studies, since these molecules appear to be unmodified at the gastric level. Conversely, the positive effect of EA has been widely demonstrated, and the effect is corroborated by other studies performed on other plants: ethanolic extract from *Ficus glomerata* fruit (FGE) contained 0.36% w/w of EA and showed significant dose-dependent anti-ulcerogenic effect in different models of induced gastritis (pylorus ligation, ethanol, and cold stress) [69]; moreover, the hydroalcoholic extract of *Anogeissus latifolia* (50% alcohol) containing 0.25% w/w of EA has been shown to possess gastroprotective activity [70] due to the presence of EA. In addition, methanol stem bark extract of *Lafoensia pacari* containing 23.4% of EA showed gastroprotective and ulcer-healing effects in animal models strictly associated to the presence of great amounts of EA in the extract [71], and an improvement of the gastric symptoms in patients with *H. pylori* gastritis was observed [72]. The mechanism of action by which EA shows antiulcer activity is partially attributed to the inhibitory effect on the gastric H^+^, K^+^-ATPase, in addition to the anti-*H. pylori* activity [38]. 

Unfortunately, no clinical studies coping with the anti-inflammatory activity of PG at the gastric level have been found, thus suggesting that the effect of the extracts and individual compounds in this area need to be elucidated. In particular, it is necessary to draw clinical trials considering the effects of PG extracts in patients with *H. pylori*-induced gastritis, alone or in combination with antibiotics.

Different preparations of PG, including extracts from peels, flowers, and seeds, in addition to the juice, show a significant anti-inflammatory activity in the gut. From all the studies taken into consideration in the present paper, some conclusions can be drawn. First of all, the pure compounds occurring in PG fruits seem to act through different pathways. Oil derived from PG seeds and its major component PuA could inhibit the expression of proinflammatory cytokines (such as IL-6, IL-8, IL-23, IL-12, and TNF-*α*) through the modulation of PPAR-*γ* and -*δ*. This is not true for PG peel extracts, as well as their components punicalagins and EA, since they do not show any effect on PPAR signalling; conversely, the main effect is due to the inhibition of the expression and secretion of several inflammatory mediators (i.e., IL-6, IL-8, MCP-1, iNOS, COX-2, and PGE_2_). The inhibitory effect is ascribed to the inhibition of the NF-*κ*B pathway and involves the MAPKs system as well. This effect was confirmed both *in vitro* and *in vivo* for the extracts and the pure compound punicalagin, while contradictory results were found for EA, since it seems to be effective only in studies performed* in vivo*. This might be explained considering the metabolic fate of PG phenolic compounds. In fact, different studies demonstrate a strong interaction between gut microbiota and PG polyphenols (i.e., EA) that are metabolized by intestinal microflora to urolithins. These metabolites themselves could modulate gut microbiota, enhancing the growth of beneficial strains in spite of pathogenic ones. Among urolithins, urolithins A has been shown to possess a significant anti-inflammatory activity both *in vitro* and *in vivo*, thus suggesting that this compound, and not its precursor EA, could be the main responsible for the anti-inflammatory properties observed with PG extracts in the gut. However it has been also showed that the inflammatory status alters the composition of intestinal microbiota, changing its metabolic capacity and the bioavailability of phenolic compounds [60]. This statement is corroborated by observation that, after consumption of PG extract, the phenolic profile of faeces obtained from healthy and DSS-fed rats is deeply changed: in normal conditions EA and punicalagin are completely metabolized to urolithins A, whereas in inflammatory conditions they can be found unmodified in the colon [60]. This suggests that biological effects of urolithins, and consequently PG, could be strictly related to the composition of individual microbiota and to the intestinal inflammatory status. For this reason, although the studies reported herein seem to recommend PG consumption to prevent or treat gastrointestinal inflammation, future clinical studies on anti-inflammatory activity at the gastrointestinal tract are necessary to clarify the beneficial effects of PG for human health. 

## References

[1] Jurenka J (2008). Therapeutic applications of pomegranate (*Punica granatum* L.): a review. *Alternative Medicine Review*.

[2] Dell’Agli M, Galli GV, Corbett Y (2009). Antiplasmodial activity of *Punica granatum* L. fruit rind. *Journal of Ethnopharmacology*.

[3] Dell’Agli M, Galli GV, Bulgari M (2010). Ellagitannins of the fruit rind of pomegranate (*Punica granatum*) antagonize in vitro the host inflammatory response mechanisms involved in the onset of malaria. *Malaria Journal*.

[4] Johanningsmeier SD, Harris GK (2011). Pomegranate as a functional food and nutraceutical source. *Annual Review of Food Science Technology*.

[5] Faria A, Conceição C (2011). The bioactivity of pomegranate: impact on health and disease. *Critical Reviews in Food Science and Nutrition*.

[6] Crabtree JE, Peichl P, Wyatt JI, Stachl U, Lindley IJD (1993). Gastric interleukin-8 and IgA IL-8 autoantibodies in *Helicobacter pylori* infection. *Scandinavian Journal of Immunology*.

[7] Crabtree JE, Xiang Z, Lindley IJD, Tompkins DS, Rappuoli R, Covacci A (1995). Induction of interleukin-8 secretion from gastric epithelial cells by a cagA negative isogenic mutant of *Helicobacter pylori*. *Journal of Clinical Pathology*.

[8] Yasumoto K, Okamoto SI, Mukaida N, Murakami S, Mai M, Matsushima K (1992). Tumor necrosis factor *α* and interferon *γ* synergistically induce interleukin 8 production in a human gastric cancer cell line through acting concurrently on AP-1 and NF-kB-like binding sites of the interleukin 8 gene. *Journal of Biological Chemistry*.

[9] Caruso R, Fina D, Peluso I (2007). IL-21 is highly produced in *Helicobacter pylori*-infected gastric mucosa and promotes gelatinases synthesis. *Journal of Immunology*.

[10] Romier B, Schneider YJ, Larondelle Y, During A (2009). Dietary polyphenols can modulate the intestinal inflammatory response. *Nutrition Reviews*.

[11] Al-Ashy R, Chakroun I, El-Sabban ME, Homaidan FR (2006). The role of NF-*κ*B in mediating the anti-inflammatory effects of IL-10 in intestinal epithelial cells. *Cytokine*.

[12] Lansky EP, Newman RA (2007). *Punica granatum* (pomegranate) and its potential for prevention and treatment of inflammation and cancer. *Journal of Ethnopharmacology*.

[13] Afaq F, Adhami VM, Mukhtar H (2005). Photochemoprevention of ultraviolet B signaling and photocarcinogenesis. *Mutation Research*.

[14] Heber D, Benzie IFF, Wachtel-Galor S (2011). Pomegranate ellagitannins. *Herbal Medicine: Biomolecular and Clinical Aspects*.

[15] Gil MI, Tomas-Barberan FA, Hess-Pierce B, Holcroft DM, Kader AA (2000). Antioxidant activity of pomegranate juice and its relationship with phenolic composition and processing. *Journal of Agricultural and Food Chemistry*.

[16] Quideau S (2009). *Chemistry and Biology of Ellagitannins*.

[17] Seeram NP, Lee R, Heber D (2004). Bioavailability of ellagic acid in human plasma after consumption of ellagitannins from pomegranate (*Punica granatum* L.) juice. *Clinica Chimica Acta*.

[18] Seeram NP, Zhang Y, McKeever R (2008). Pomegranate juice and extracts provide similar levels of plasma and urinary ellagitannin metabolites in human subjects. *Journal of Medicinal Food*.

[19] Cerdá B, Espín JC, Parra S, Martínez P, Tomás-Barberán FA (2004). The potent in vitro antioxidant ellagitannins from pomegranate juice are metabolised into bioavailable but poor antioxidant hydroxy-6H-dibenzopyran-6-one derivatives by the colonic microflora of healthy humans. *European Journal of Nutrition*.

[20] Pérez-Vicente A, Gil-Izquierdo A, García-Viguera C (2002). In vitro gastrointestinal digestion study of pomegranate juice phenolic compounds, anthocyanins, and vitamin C. *Journal of Agricultural and Food Chemistry*.

[21] McDougall GJ, Fyffe S, Dobson P, Stewart D (2005). Anthocyanins from red wine—their stability under simulated gastrointestinal digestion. *Phytochemistry*.

[22] McGhie TK, Walton MC (2007). The bioavailability and absorption of anthocyanins: towards a better understanding. *Molecular Nutrition and Food Research*.

[23] Aura AM, Martin-Lopez P, O’Leary KA (2005). In vitro metabolism of anthocyanins by human gut microflora. *European Journal of Nutrition*.

[24] Fleschhut J, Kratzer F, Rechkemmer G, Kulling SE (2006). Stability and biotransformation of various dietary anthocyanins in vitro. *European Journal of Nutrition*.

[25] Saniee P, Hajimahmoodi M, Foroumadi P (2009). Antibacterial activity of plant extracts against *H. pylori*. *Helicobacter*.

[26] Hajimahmoodi M, Shams-Ardakani M, Saniee P (2011). In vitro antibacterial activity of some Iranian medicinal plant extracts against *Helicobacter pylori*. *Natural Product Research*.

[27] Voravuthikunchai SP, Mitchell H (2008). Inhibitory and killing activities of medicinal plants against multiple antibiotic-resistant *Helicobacter pylori*. *Journal of Health Science*.

[28] Oates PJ, Hakkinen JP (1988). Studies on the mechanism of ethanol-induced gastric damage in rats. *Gastroenterology*.

[29] Ajaikumar KB, Asheef M, Babu BH, Padikkala J (2005). The inhibition of gastric mucosal injury by *Punica granatum* L. (pomegranate) methanolic extract. *Journal of Ethnopharmacology*.

[30] Alam MS, Alam MA, Ahmad S, Najmi AK, Asif M, Jahangir T (2010). Protective effects of *Punica granatum* in experimentally-induced gastric ulcers. *Toxicology Mechanisms and Methods*.

[31] Lai S, Zhou Q, Zhang Y, Shang J, Yu T (2009). Effects of Pomegranate tannins on experimental gastric damages. *Zhongguo Zhongyao Zazhi*.

[32] Gharzouli K, Khennouf S, Amira S, Gharzouli A (1999). Effects of aqueous extracts from *Quercus ilex* L. root bark, *Punica granatum* L. fruit peel and Artemisia herba-alba Asso leaves on ethanol-induced gastric damage in rats. *Phytotherapy Research*.

[33] Beserra AMSES, Calegari PI, Souza MDC (2011). Gastroprotective and ulcer-healing mechanisms of ellagic acid in experimental rats. *Journal of Agricultural and Food Chemistry*.

[34] Fiorucci S, Antonelli E, Morelli A (2001). Mechanism of non-steroidal anti-inflammatory drug-gastropathy. *Digestive and Liver Disease*.

[35] Hawkey CJ (1989). Prostaglandins: mucosal protection and peptic ulceration. *Methods and Findings in Experimental and Clinical Pharmacology*.

[36] Chatterjee A, Chatterjee S, Das S (2012). Ellagic acid facilitates indomethacin-induced gastric ulcer healing via COX-2 up-regulation. *Acta Biochimica et Biophysica Sinica*.

[37] Gautam R, Sharma SC (2012). Anti-ulcer activity of *Punica granatum* L. in diabetic rats. *International Journal of Pharmacy and Pharmaceutical Sciences*.

[38] Murakami S, Isobe Y, Kijima H, Nagai H, Muramatu M, Otomo S (1991). Inhibition of gastric H+,K+-ATPase and acid secretion by ellagic acid. *Planta Medica*.

[39] Iino T, Tashima K, Umeda M (2002). Effect of ellagic acid on gastric damage induced in ischemic rat stomachs following ammonia or reperfusion. *Life Sciences*.

[40] Adams LS, Seeram NP, Aggarwal BB, Takada Y, Sand D, Heber D (2006). Pomegranate juice, total pomegranate ellagitannins, and punicalagin suppress inflammatory cell signaling in colon cancer cells. *Journal of Agricultural and Food Chemistry*.

[41] Romier-Crouzet B, van de Walle J, During A (2009). Inhibition of inflammatory mediators by polyphenolic plant extracts in human intestinal Caco-2 cells. *Food and Chemical Toxicology*.

[42] Hollebeeck S, Winand J, Herent MF (2012). Anti-inflammatory effects of pomegranate (*Punica granatum* L.) husk ellagitannins in Caco-2 cells, an in vitro model of human intestine. *Food & Function*.

[43] Rastall RA, Gibson GR, Gill HS (2005). Modulation of the microbial ecology of the human colon by probiotics, prebiotics and synbiotics to enhance human health: an overview of enabling science and potential applications. *FEMS Microbiology Ecology*.

[44] Bialonska D, Kasimsetty SG, Schrader KK, Ferreira D (2009). The effect of pomegranate (*Punica granatum* l.) byproducts and ellagitannins on the growth of human gut bacteria. *Journal of Agricultural and Food Chemistry*.

[45] Seeram NP, Henning SM, Zhang Y, Suchard M, Li Z, Heber D (2006). Pomegranate juice ellagitannin metabolites are present in human plasma and some persist in urine for up to 48 hours. *Journal of Nutrition*.

[46] Bialonska D, Ramnani P, Kasimsetty SG, Muntha KR, Gibson GR, Ferreira D (2010). The influence of pomegranate by-product and punicalagins on selected groups of human intestinal microbiota. *International Journal of Food Microbiology*.

[47] Dobroslawa B, Kasimsetty SG, Khan SI, Daneel F (2009). Urolithins, intestinal microbial metabolites of pomegranate ~ellagitannins, exhibit potent antioxidant activity in a cell-based assay. *Journal of Agricultural and Food Chemistry*.

[48] Sergent T, Piront N, Meurice J, Toussaint O, Schneider YJ (2010). Anti-inflammatory effects of dietary phenolic compounds in an in vitro model of inflamed human intestinal epithelium. *Chemico-Biological Interactions*.

[49] González-Sarrías A, Espín JC, Tomás-Barberán FA, García-Conesa MT (2009). Gene expression, cell cycle arrest and MAPK signalling regulation in Caco-2 cells exposed to ellagic acid and its metabolites, urolithins. *Molecular Nutrition and Food Research*.

[50] González-Sarrías A, Larrosa M, Toms-Barberán FA, Dolara P, Espín JC (2010). NF-*κ*B-dependent anti-inflammatory activity of urolithins, gut microbiota ellagic acid-derived metabolites, in human colonic fibroblasts. *British Journal of Nutrition*.

[51] Gimenez-Bastida JA, Larrosa M, Gonzalez-Sarrias A (2012). Intestinal ellagitannin metabolites ameliorate cytokine-induced inflammation and associated molecular markers in human colon fibroblasts. *Journal of Agricultural and Food Chemistry*.

[52] Ogawa Y, Kanatsu K, Iino T (2002). Protection against dextran sulfate sodium-induced colitis by microspheres of ellagic acid in rats. *Life Sciences*.

[53] Singh K, Jaggi AS, Singh N (2009). Exploring the ameliorative potential of *Punica granatum* in dextran sulfate sodium induced ulcerative colitis in mice. *Phytotherapy Research*.

[54] Umesalma S, Sudhandiran G (2010). Differential inhibitory effects of the polyphenol ellagic acid on inflammatory mediators NF-*κ*B, iNOS, COX-2, TNF-*α*, and IL-6 in 1,2-dimethylhydrazine-induced rat colon carcinogenesis. *Basic and Clinical Pharmacology and Toxicology*.

[55] Rosillo MA, Sanchez-Hidalgo M, Cárdeno A, de la Lastra CA (2011). Protective effect of ellagic acid, a natural polyphenolic compound, in a murine model of Crohn's disease. *Biochemical Pharmacology*.

[56] Rosillo MA, Sanchez-Hidalgo M, Cardeno A (2012). Dietary supplementation of an ellagic acid-enriched pomegranate extract attenuates chronic colonic inflammation in rats. *Pharmacological Research*.

[57] Lian J, Ding W, Sun JX, Lü XM (2009). Experimental study of aqueous extract of *Pericarpium granati* for treating ulcerative colitis of rats. *Pharmaceutical Care and Research*.

[58] Neyrinck AM, van Hee VF, Bindels LB (2012). Polyphenol-rich extract of pomegranate peel alleviates tissue inflammation and hypercholesterolaemia in high-fat diet-induced obese mice: potential implication of the gut microbiota. *British Journal of Nutrition*.

[59] Khokhlova EV, Smeianov VV, Efimov BA (2012). Anti-inflammatory properties of intestinal Bifidobacterium strains isolated from healthy infants. *Microbiology and Immunology*.

[60] Larrosa M, González-Sarrías A, Yáñez-Gascón MJ (2010). Anti-inflammatory properties of a pomegranate extract and its metabolite urolithin-A in a colitis rat model and the effect of colon inflammation on phenolic metabolism. *Journal of Nutritional Biochemistry*.

[61] Nagao K, Yanagita T (2005). Conjugated fatty acids in food and their health benefits. *Journal of Bioscience and Bioengineering*.

[62] Hwang DM, Kundu JK, Shin JW, Lee JC, Lee HJ, Surh YJ (2007). cis-9,trans-11-Conjugated linoleic acid down-regulates phorbol ester-induced NF-*κ*B activation and subsequent COX-2 expression in hairless mouse skin by targeting I*κ*B kinase and PI3K-Akt. *Carcinogenesis*.

[63] Boussetta T, Raad H, Lettéron P (2009). Punicic acid a conjugated linolenic acid inhibits TNF*α*-induced neutrophil hyperactivation and protects from experimental colon inflammation in rats. *PLoS ONE*.

[64] Bassaganya-Riera J, DiGuardo M, Climent M (2011). Activation of PPAR*γ* and *δ* by dietary punicic acid ameliorates intestinal inflammation in mice. *British Journal of Nutrition*.

[65] Katayama K, Wada K, Nakajima A (2003). A novel PPAR*γ* gene therapy to control inflammation associated with inflammatory bowel disease in a murine model. *Gastroenterology*.

[66] Coursodon-Boyiddle CF, Snarrenberg CL, Adkins-Rieck CK (2012). Pomegranate seed oil reduces intestinal damage in a rat model of necrotizing enterocolitis. *American Journal of Physiology*.

[67] Hasan R, Hossain M, Akter R (2009). Antioxidant, antidiarrhoeal and cytotoxic properties of *Punica granatum* Linn.. *Latin American Journal of Pharmacy*.

[68] Qnais EY, Elokda AS, Ghalyun YYA, Abdulla FA (2007). Antidiarrheal activity of the aqueous extract of *Punica granatum* (pomegranate) peels. *Pharmaceutical Biology*.

[69] Rao CV, Verma AR, Vijayakumar M, Rastogi S (2008). Gastroprotective effect of standardized extract of *Ficus glomerata* fruit on experimental gastric ulcers in rats. *Journal of Ethnopharmacology*.

[70] Govindarajan R, Vijayakumar M, Singh M (2006). Antiulcer and antimicrobial activity of *Anogeissus latifolia*. *Journal of Ethnopharmacology*.

[71] Tamashiro-Filho P, Sikiru Olaitan B, Tavares de Almeida DA (2012). Evaluation of antiulcer activity and mechanism of action of methanol stem bark extract of Lafoensia pacari A. St.-Hil. (Lytraceae) in experimental animals. *Journal of Ethnopharmacology*.

[72] Da Mota Menezes V, Atallah ÁN, Lapa AJ, Catapani WR (2006). Assessing the therapeutic use of Lafoensia pacari St. Hil. extract (Mangava-Brava) in the eradication of *Helicobacter pylori*: double-blind randomized clinical trial. *Helicobacter*.

